# Assessment of a combined musculoskeletal and chest deep learning-based detection solution in an emergency setting

**DOI:** 10.1016/j.ejro.2023.100482

**Published:** 2023-03-10

**Authors:** Alexandre Parpaleix, Clémence Parsy, Marina Cordari, Mehdi Mejdoubi

**Affiliations:** a Department of Radiology, Valenciennes General Hospital, Valenciennes, France; b Arterys Inc., San Francisco, CA, USA

**Keywords:** Deep learning, Emergency, Xray, Musculoskeletal, Chest, Add-on

## Abstract

**Rationale and objectives:**

Triage and diagnostic deep learning-based support solutions have started to take hold in everyday emergency radiology practice with the hope of alleviating workflows. Although previous works had proven that artificial intelligence (AI) may increase radiologist and/or emergency physician reading performances, they were restricted to finding, bodypart and/or age subgroups, without evaluating a routine emergency workflow composed of chest and musculoskeletal adult and pediatric cases. We aimed at evaluating a multiple musculoskeletal and chest radiographic findings deep learning-based commercial solution on an adult and pediatric emergency workflow, focusing on discrepancies between emergency and radiology physicians.

**Material and methods:**

This retrospective, monocentric and observational study included 1772 patients who underwent an emergency radiograph between July and October 2020, excluding spine, skull and plain abdomen procedures. Emergency and radiology reports, obtained without AI as part of the clinical workflow, were collected and discordant cases were reviewed to obtain the radiology reference standard. Case-level AI outputs and emergency reports were compared to the reference standard. DeLong and Wald tests were used to compare ROC-AUC and Sensitivity/Specificity, respectively.

**Results:**

Results showed an overall AI ROC-AUC of 0.954 with no difference across age or body part subgroups. Real-life emergency physicians’ sensitivity was 93.7 %, not significantly different to the AI model (*P* = 0.105), however in 172/1772 (9.7 %) cases misdiagnosed by emergency physicians. In this subset, AI accuracy was 90.1 %.

**Conclusion:**

This study highlighted that multiple findings AI solution for emergency radiographs is efficient and complementary to emergency physicians, and could help reduce misdiagnosis in the absence of immediate radiological expertize.

## Highlights

• The overall AI performance was invariant across age and bodypart subgroups.
• The AI sensitivity was not significantly different from emergency physicians’ one (92,9 % vs 93,7 %; *P* = 0.105).
• The AI accuracy was 90.1 % on a subgroup of difficult cases misdiagnosed at the emergency department.

## 1 Introduction

In the past decades, workflow pressure in emergency departments (EDs) have increased worldwide [1] with a concurrent increase in requested medical imaging volumes 24 h a day, 7 days a week, while most radiology departments struggle to provide resources for 24/7 coverage. Consequently, ED physicians are facing the need to interpret the radiographic examinations prior to the availability of a radiology report, raising new organizational challenges to maintain diagnosis accuracy and rapid report turnaround time [2]. In this context, artificial intelligence (AI), and more precisely deep learning applied to radiological imaging, has emerged as an application to help improve the ED workflow [3], [4], with most of the commercial solutions focusing on triage and diagnosis of chest or musculoskeletal (MSK) plain radiographs [5].

Improvements of emergency physicians’ and/or radiology residents’ diagnosis performances for appendicular skeletal fractures detection [6], [7], [8] or chest abnormalities detection [9], [10] have been evidenced in several studies conducted in ED settings. However, their impact on a whole emergency workflow remains unclear, as they focus on single imaging finding, bodypart or age group [11], [12].

Therefore, the main objective of the study was to assess the performance of a deep learning-based commercial solution to triage an adult and pediatric emergency workflow by detecting multiple MSK and chest radiographic findings. Secondarily, we assessed its impact on discrepancies between emergency physicians and radiologists.

## 2 Materials and methods

This retrospective, monocentric and observational study received nonfinancial support by Milvue and Arterys Inc. which provided the AI model and the cloud-based infrastructure to run it, respectively. All authors had control of the data and information submitted for publication. Study was approved by the IRB committee (CRM-2204-262). We followed the Checklist for AI in Medical Imaging [13].

We retrieved radiographic studies and their corresponding radiology and ED reports of consecutive adult and pediatric patients from the emergency department of our institution between July and October 2020. All radiological, clinical procedures and reports were performed without the use of any AI-based solution, as part of the local standard clinical routine by 31 radiologists and 26 ED physicians having full medical record access. Consistent with the most observed workflow in French EDs, emergency physicians first interpreted x-ray exams allowing for the patient's immediate management and a radiologist later reported them in a maximum time set of 12 h during working days and 24 h during the weekend.

We excluded: i) any radiological examination not associated with both an ED and a radiology report; and ii) radiographic examination imaging of the spine, skull and plain abdomen since they were not supported by the AI software.

A radiology resident reviewed all ED and radiology reports to classify them as positive, negative or in doubt to the presence of any of the 7 following radiological findings at the case-level: i) fracture; ii) dislocation; iii) elbow joint effusion; iv) pneumothorax; v) pleural effusion; vi) lung parenchymal opacity; vii) lung nodule. Cases of mismatch between the ED and radiology reports constituted the discordant population subgroup and were reviewed by a senior musculoskeletal radiologist (12 years of experience) for adjudication on discordant cases, with access to all necessary and available complementary data of the clinical records but not the AI. Post-adjudication radiology report-derived labels were binarized at the case-level, being positive if any finding was detected positive or doubt on one or multiple images of the series and/or studies, and served as the reference standard (ground-truth) for data analysis.

All included radiological examinations were locally anonymized and uploaded to a cloud-based radiology platform (Arterys Inc., USA) for stand-alone post-processing by a deep learning model trained on 121,808 annotated images from 4 French radiology centers, excluding our institution (Milvue Suite v1.8, Milvue, France). Each study was processed for detecting the presence, doubt or absence of any of the seven above-mentioned imaging findings. Output was binarized at the accession number-level (i.e. case-level), using the same protocol as for reports binarization.

Descriptive statistics are provided as absolute numbers and percentage for categorical variables, while continuous variables are presented as mean and standard deviation (SD) or median and interquartile range (IQR) depending on data distribution. The Shapiro-Wilk test was used for normality distribution check. AI performance metrics were included: area under the receiver operating characteristic curve (ROC-AUC), accuracy, sensitivity (Se), specificity (Sp), negative predictive value (NPV) and positive predictive value (PPV). 95 % confidence interval (95 % CI) of ROC-AUC was computed with 2000 stratified bootstrap replicates. AI performances were compared to the ED physicians’ ones following the protocol suggested by Roldán-Nofuentes in 2020 [14]. Sub-group analysis was performed for relevant age groups and bodypart groups: i) pediatric subjects (< 18 years); adult population (18–64 years); geriatric population (> 64 years); ii) upper limbs, lower limbs, thorax and multiple bodyparts. Unpaired DeLong tests [15] were used to compare AUC values. Statistical significance was set to 0.05 and Bonferroni correction was used to account for multiple comparisons. Statistical analyses were performed using multiple statistical software (Python, v.3.7.0 and R, version 4.2.1).

## 3 Results

**Fig. 1:**
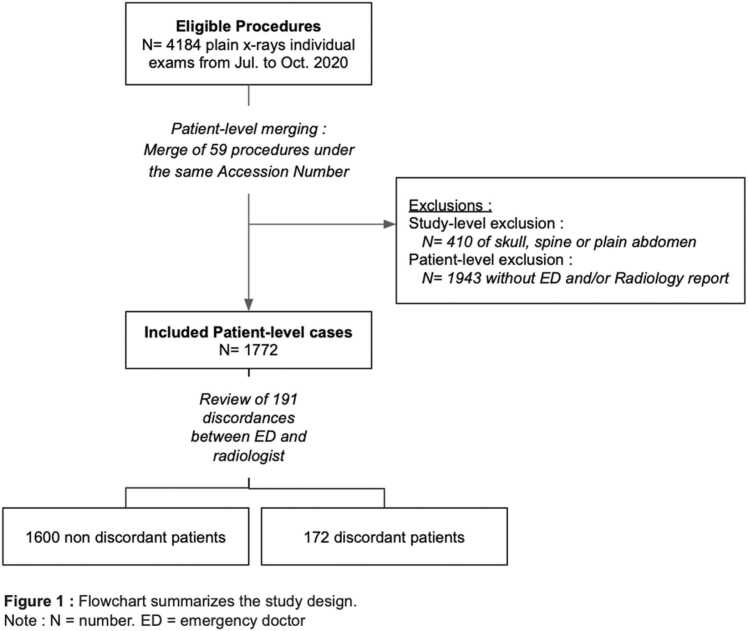
**Flowchart summarizes the study design.** Note: N = number. ED = emergency doctor.

**Fig. 2:**
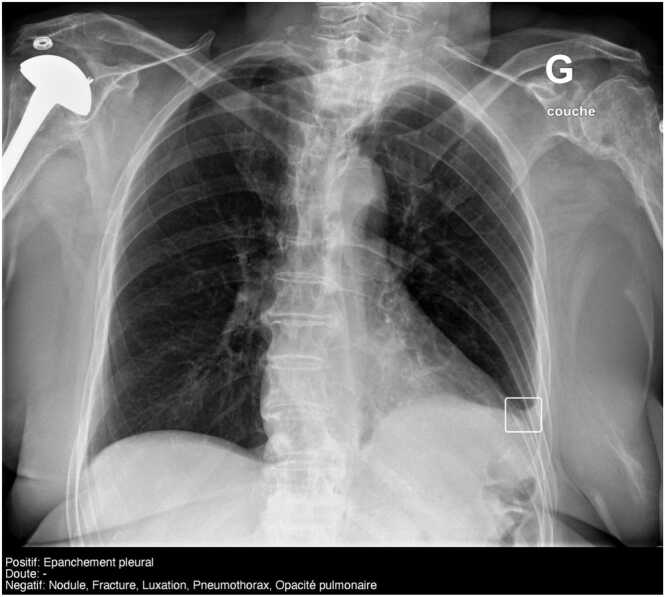
**Example of a chest discordant case correctly diagnosed by AI.** Frontal view of chest radiograph in adult, as part of a multiple bodyparts examinations (including hip and knee, reported as normal). The chest was reported as normal by the ED. The radiologist reported a minimal left pleural effusion. AI output shows positive pleural effusion (white solid bounding box). French translation: doute = doubt; opacité pulmonaire = lung parenchymal opacity; luxation = dislocation; negatif = negative; positif = positive.

**Fig. 3:**
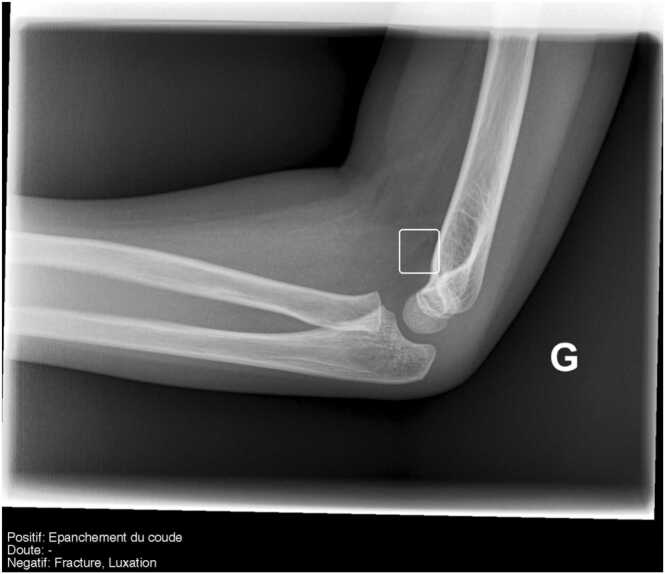
**Example of an MSK discordant case correctly diagnosed by AI.** Lateral view of an elbow radiograph in a child, reported as normal by the ED. The radiologist reported a joint effusion without visible fracture. AI output highlights the anterior elbow fat pad as the sign of the joint effusion. French translation: doute = doubt; luxation = dislocation; negatif = negative; positif = positive.

**Fig. 4:**
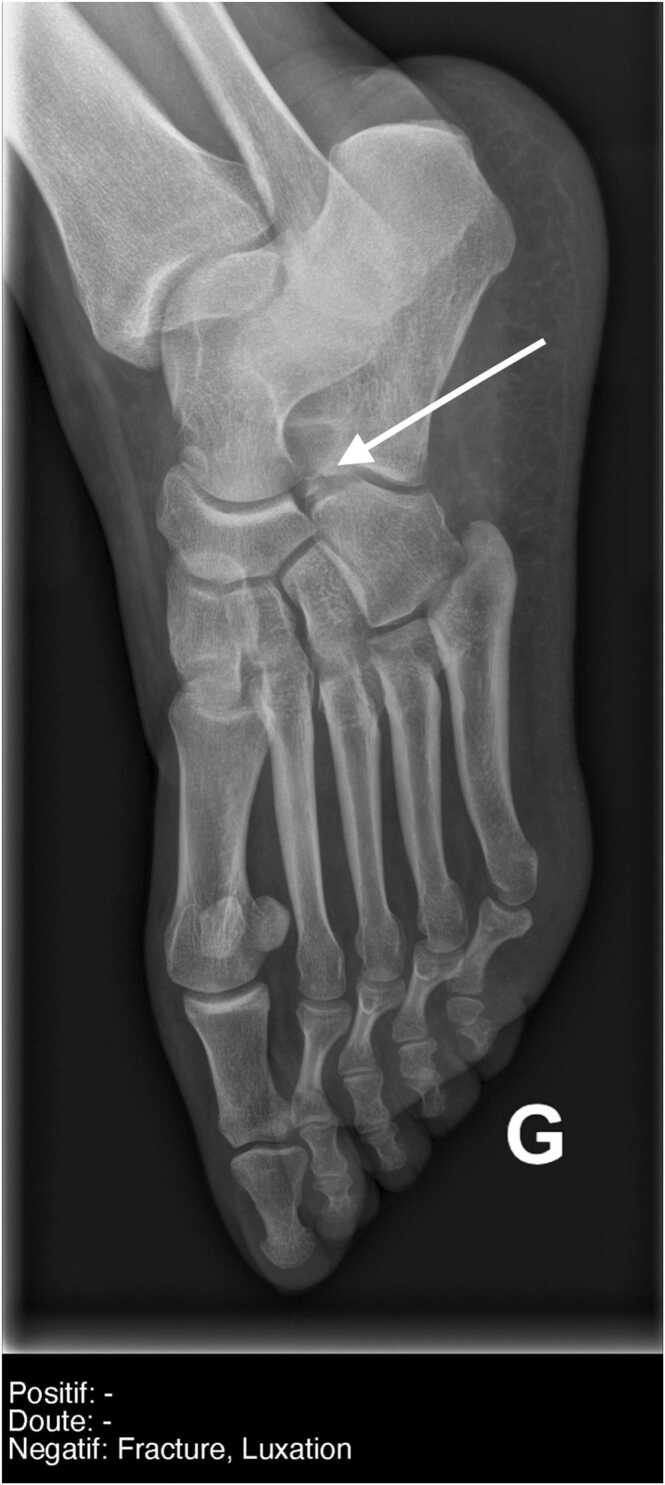
**Example of an MSK discordant case missed by AI.** 3/4 view of a foot radiograph in an adult patient, reported as normal by the ED and the AI. The radiologist reported an avulsion fracture of the calcaneal rostrum only visible on the 3⁄4 view, related to a Chopart joint sprain (white arrow). French translation: doute = doubt; luxation = dislocation; negatif = negative; positif = positive.

Both ED and radiological reports were available in 1772/3715 (47.7 %) patients (Fig. 1). Details of ages, number of pathological cases and bodyparts distribution are provided in Table 1 and Supplemental Table 1. Before binarization, 15/1772 (0.8 %) of overall cases were reported as doubtful by the radiologist. Among them, 9/15 (60 %) were discordant cases, 14/15 were considered abnormal after expert review, and AI achieved a 10/15 (66.7 %) classification accuracy (Fig. 2, Fig. 3, Fig. 4).

**Table 1:** Study sample and discordant subgroup characteristics.

	Overall population (1772 cases)		Discordant subgroup (172 cases)	
Age groups	Age (years)*	No. Of positive cases (%)	Age (years)*	No. Of positive cases (%)
Total	30 (16–52)	616/1772 (34.8)	29 (16–48)	39/172 (22.7)
Pediatrics (< 18 y)	10 (6–13)	173/478 (36.2)	11 (7.25–14)	6/50 (12.0)
Adults (18–65 y)	34 (24–47)	283/1003 (28.2)	34 (29.5–47)	26/103 (25.2)
Geriatrics (≥ 65 y)	81 (70–87)	160/291 (55.0)	79 (71–88,5)	7/19 (36.8)

Note: *ages were not normally distributed (Shapiro-Wilk test, p < 0.05), expressed as median. 25th and 75th percentile values are in parentheses Otherwise, data are numbers of patients with percentages in parentheses.

**Fig. 5:**
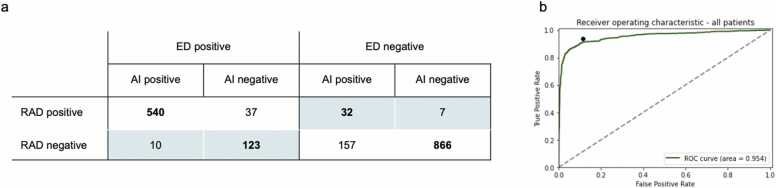
**AI added value and stand-alone performance on the total included population.** 1a – Confusion matrix of all binarized results for AI, radiologists and emergency physicians. Doctors and radiologists disagree in 17 + 155 = 172 cases (colored background). AI agrees with radiologists in 1406 + 155 = 1561 cases (text in bold). 1b – ROC curve of the AI model (green). The emergency physician Se/Sp operating point is displayed as a black dot. Note: RAD = radiologist. AI = Artificial Intelligence solution. ED: emergency physician.

After reviewing discordant cases by a senior MSK radiologist, 18/190 (9.4 %) of radiological diagnosis were modified, with 10/191 (5.2 %) from negative to positive and 8/191 (4.2 %) from positive to negative. In view of this corrected reference standard, the interpretation by the emergency physician was considered a misdiagnosis in 172/1772 (9.7 %) patients, on which AI achieved a 90.1 % classification accuracy (Fig. 5).

Stand-alone AI performance analysis assessed an overall ROC-AUC of 0.954 [0.942–0.965 – 95 % CI]. Before binarization, 11.7 % of overall cases were classified as doubtful by the AI. Se, Sp, NPV and PPV values were 92.8 %, 85.6 %, 95.7 % and 77.4 % respectively (Table 2). The lowest and highest AI ROC-AUC values for bodyparts were reported in the thorax subgroup (0.895 [0.834–0.955 - 95 % CI]), and the upper limbs subgroup (0.97 [0.957–0.982 - 95 % CI]), respectively. Regarding ages, ROC-AUC values for adults were the highest (0.958 [0.942–0.973 – 95 %CI]) and geriatrics the lowest (0.927 [0.897–0.957 - 95 % CI]). There was no significant difference in AI ROC-AUC value across all age nor bodyparts subgroups (unpaired DeLong test, Supplemental Fig. 1 and Table 2). No significant differences were found between AI standalone and emergency physicians’ performances (paired Wald test, see Supplemental Results). In the 211/1772 incorrect AI outputs, emergency physicians achieved a 91.9 % classification accuracy. Among these cases, and without the use of AI, the interpretation of radiologists and emergency physicians disagreed in 17 cases, i.e. 8 % of the discordant cases and 0.96 % of all the cases. 7 were false negatives and 10 were false positives.

**Table 2:** Detailed performance in investigated subgroups.

	Overall population (1772 cases)				Discordant subgroup (172 cases)
Parameters	Total	Pediatrics	Adults	Geriatrics	Total
**ROC-AUC**					
- AI model	0.954	0.954	0.958	0.927	0.872
[95 %CI]	[0.942;0.965]	[0.934;0.975]	[0.942;0.973]	[0.897;0.957]	[0.792;0.952]
- ED report	NA	NA	NA	NA	NA
**Sensitivity**					
- AI model	92.9	93.6	91.9	93.8	82.1
- ED report	93.7	96.5	90.8	95.6	NA
**Specificity**					
- AI model	85.6	84.6	87.8	75.6	92.5
- ED report	88.5	85.6	89.3	90.8	NA
**NPV**					
- AI model	95.7	95.9	96.5	90.8	94.6
- ED report	96.3	97.8	96.1	94.4	NA
**PPV**					
- AI model	77.4	77.5	74.7	82.4	76.2
- ED report	81.3	79.1	76.9	92.7	NA

Data are percentage, otherwise mentioned.

Note: AI = Artificial Intelligence. ED = emergency doctor, NA: not applicable; ROC-AUC: area under the curve-receiver operating characteristic.

## 4 Discussion

Plain radiographs are the most frequent radiological procedures prescribed in ED. Obtaining an immediate radiological interpretation is out of reach on a 24/7 basis. AI may help to efficiently prioritize the need of immediate radiological interpretation, which could alleviate workflow constraints and optimize patient’s management. In our study, based on real life external clinical data, we assessed the stand-alone performance of a deep learning-based commercial solution in a routine adult and pediatric emergency radiographic workflow. The overall AI performance was invariant across age and bodypart subgroups, and sensitivity was not significantly different from emergency physicians’ (*P* = 0.105), although its accuracy was 90.1 % on the 9.7 % challenging cases misdiagnosed at the emergency department.

The diagnostic performances of the AI model compared favorably with that of previously published deep learning algorithms for radiographic anomaly detection. To our knowledge, all of previous work focused on a limited spectrum of bodyparts, ages and/or findings [10], [11], [16], [17], [18], [19]. A strength was therefore to combine MSK and chest anomaly detection, allowing for a wider range of cases to be covered in the emergency radiographic workflow, excluding only views of the spine, skull and abdomen. In addition, no significant difference in performance was found across age nor bodypart subgroups which is of importance for the widespread use of AI in the daily clinical environment. To our knowledge, geriatric population has not been addressed in previous works, although elderly patients are increasingly over-represented in emergency workflows [1], with diagnostic challenges due to osteopenia and arthrosis. Our study’s retrospective design, with study sample extracted from a routine workflow, enabled to limit context bias and the Hawthorne effect. In this light, we further found that the use of AI by emergency physicians could have reduced misdiagnosis in ED with potential impact on patient management if AI’s results would have been followed when deemed necessary. This result compares to studies demonstrating an increase in emergency physician’s diagnostic performance with AI [16], [17]. Recently, AI was proven to increase emergency physicians’ sensitivity by 13 %, with a stand-alone sensitivity of 61.3 % [6]. Interestingly, and as a strength of this study, we reported a stand-alone emergency physicians’ sensitivity of 93.7 % in real-world conditions where all physicians have access to clinical information [20], [21], establishing the true performance of emergency physicians in real-life. In addition, although the overall performance of the AI was not found to be superior to that of the emergency physician, the potential impact on reducing misdiagnosis is promising, indicating that the AI and emergency physicians did not fail on the same cases, highlighting their expected synergy. Given the high NPV of the AI solution and the high recall rate for misdiagnosis, we foresee the implementation of an AI-based 24/7/365 emergency radiographic workflow without replacing radiological interpretation but rather securing patient management through its use as both a rule-out (triage) and a computer-aided diagnosis (add-on) solution [22], [23]. Doubtful exams as classified by AI and/or after radio-clinical assessment, could be prioritized for radiologist review. Exams not raising any diagnostic concern could be reviewed later. Under this hypothesis, AI could alleviate emergency radiographic workflow constraints.

One of the major concerns about the implementation of AI in daily routine is the possibility of an AI-induced misdiagnosis by the healthcare professional, radiologist, or emergency physician, who was initially right without AI. For emergency physicians, their specificity with AI aid was not found significantly reduced in the recent literature for fracture detection [6], [8], [17]. Lindsey et al. demonstrated a significant increase in specificity of emergency physicians aided by AI for fracture detection [16]. For chest anomalies, Hwang et al. demonstrated a significant decrease in specificity in radiology residents aided by AI [10], whereas Sung et al. demonstrated a significant improvement of radiologists’ specificity when using AI [19], thus one can expect that false positive diagnosis by AI will be mostly corrected by the emergency physician. In particular, among the incorrect AI outputs in our study, AI and emergency physicians agreed in 17 cases, i.e. 0.96 % of all the cases. Although these cases can raise concerns for patient’s management, it was significantly lower in proportion than the AI-induced potential beneficial reduction of misdiagnosis by the emergency physician. Yet, the benefit/risk ratio was favorable for AI.

Our study had limitations. First, the study is monocentric although the number of radiologists and emergency physicians was large from this institution. Second, case-wise binarized data collection did not allow further per-finding analysis and ignored the partial detection of findings when the correct finding was indicated. Third, although many cases were excluded due to a missing report, it was due to workflow constraints rather than a specific clinical process. Finally, as per the study design, impact assessment could only be estimated as ED physician performance was not compared with and without AI, and the turnaround time was not evaluated. In particular, false positives by AI could induce uncertainty to the emergency physician with collateral consequences on diagnosis accuracy, turn around time or complementary medical imaging.

In conclusion, a combined multiple MSK and chest findings AI solution for radiographs in a routine emergency setting was proved efficient to detect multiple bone and chest radiographic findings, as well as potentially impactful to reduce the error rate in the absence of immediately available radiological expertize. Further prospective works, including medico-economic studies, are warranted.

## Declaration of Interest statement

This research did not receive any specific grant from funding agencies in the public, commercial, or not-for-profit sectors.

A.P. is the cofounder, and chairman of Milvue. M.C. is employee at Arterys Inc. C.P. and M.M. has no conflict of interest to declare. Valenciennes Hospital radiology department received nonfinancial support by Milvue and Arterys Inc. which provided the AI model and the cloud-based infrastructure to run it free of charge, respectively.

## CRediT authorship contribution statement

**A.P:** Data collection, Data curation, Analysis, Writing – original draft, Writing – review & editing. **M.C:** Validation, Data curation, Analysis, Writing – original draft, Writing – review & editing. **C.P:** Conceptualization, Methodology, Validation, Analysis, Writing – original draft, Writing – review & editing, Supervision. **M.M:** Conceptualization, Methodology, Validation, Analysis, Writing – original draft, Writing – review & editing, Supervision.

## Declaration of Competing Interest

The authors declare the following financial interests/personal relationships which may be considered as potential competing interests: Conflicts of interest are as follows: A.P. is the cofounder, and chairman of Milvue. M.C. is employee at Arterys Inc. C.P. and M.M. has no conflict of interest to declare.

## Data Availability

Data generated by the authors are available from the corresponding author upon request.
