# Supplementary material for: Assessment of a combined musculoskeletal and chest deep learning-based detection solution in an emergency setting
Source: Eur J Radiol Open. 2023 Mar 10;10:100482. doi: 10.1016/j.ejro.2023.100482 (PMC10023863; doi:10.1016/j.ejro.2023.100482)
Supplement: Supplementary file 1 — Supplementary material [file mmc1.docx]

**Supplemental Materials**

**Supplemental methods**

*Code and Raw Data Availability*

As part of a commercial software, the development dataset, the proprietary code and the weights of the AI model are not publicly available.

Clinical datasets with the corresponding reports are not publicly available, proprietary to the author’s institution organization.

*AI model*

The AI model was developed by Milvue, France, using deep learning algorithms designed for the identification and localization of abnormalities on medical radiographs. The code is based on the “TensorFlow Model Garden” framework ^21^, further revised and adjusted for emergency radiographs and multiple findings detection by Milvue.

The model received full resolution Digital imaging and communications in medicine (DICOM) radiographs as inputs and utilized a combination of convolutional neural networks (CNNs) and object detection techniques to accurately identify and locate regions of interest in the radiograph. The CNN architecture combined a Resnet backbone and a feature pyramid network (FPN) which extracted features at multiple scales. Each radiographic projection, corresponding to a single DICOM image, was independently analyzed to detect any of seven imaging findings mentioned in this research protocol. Each finding was associated with a probability score which was then automatically categorized into 3 types of output based on 2 pre-defined and fixed thresholds: negative, doubt or positive. If any, identified findings were labeled and localized with bounding boxes burnt in the pixels in the original input image, indicating its location and size on the radiograph. Depending on the probability score and therefore its category, a finding was displayed by a dashed white box on the radiograph when in doubt, and by a solid white box when positive. To build an instance or study level prediction, the aggregated prediction of all boxes in all views was considered.

*Data collection, DICOM metadata, and AI processing*

All x-rays were acquired from a Siemens-Healthineers Ysio Max machine.

DICOM metadata were parsed by the local IT PACS manager of the institution to extract StudyUID, Accession number, Age and Bodypart for data collection purposes.

All examinations from the study sample were locally anonymized and uploaded to a cloud-based radiology platform (Arterys Inc., USA) for AI inference (Chest|MSK AI app powered by Milvue, France). To binarize the software output, a study was classified as positive if any of the findings was detected (doubt or positive), on one or multiple images of the study series. More particularly, the AI probability score was defined as the maximum probability score across all findings.

The Accession number was used to merge multiple studies of the same patient (N = 59). In this view, the case was considered positive if any of the selected findings was detected in at least one radiological study. Similarly, the maximum probability score per accession number was the maximum probability score across studies.

*Statistics*Statistical analyses were performed using dedicated python or R scripts. The following R packages or scripts were used: *pROC* ^22,23; 12^. The following python packages were used: numpy (v.1.21.5 ), pandas (v.1.3.5), sklearn (v.1.0.2), seaborn (v. 0.11.2) and scipy (v. 1.7.3).

*Management of Missing Data*

There were no missing data on the overall population nor on the discordant subgroup, as per definition they contain the associated AI outputs, ED and radiology reports.

**Supplemental results**

Descriptive subgroup statistics

*By Age*

We compared ROC-AUC values across age groups on the overall population using the unpaired DeLong test. The overall AI ROC-AUC was 0.954 (0.942 – 0.965 – 95% confidence interval [CI]). It was 0.958 (0.942 – 0.973 – 95%CI) for the adults subgroup; 0.954 (0.934 – 0.975 – 95% CI) for the pediatrics subgroup and 0.927 (0.897 – 0.957 - 95% CI) for the geriatrics subgroup (Supplemental Figure 1).

There was no significant difference in AI ROC-AUC value across all age subgroups: adults vs. pediatrics (delta = 0.35%, p = 0.790); adults vs. geriatrics (delta = 3,05%, p = 0,078); pediatrics vs geriatrics (delta = 2.70%, p = 0.147). P value for significance was set to 0.017 after Bonferroni corrections.

Se, Sn, NPV and PPV of the AI-based software are summarized in Table 2 of the main manuscript for the overall population with details for each subgroup. On the discordant subgroup, Se, Sn, NPV and PPV values were respectively: 83.3%, 93.2%, 97.6% and 62.5% for the pediatrics subgroup; 84.6%, 90.9%, 94.6% and 75.9% for the adults subgroup; and 71.4%, 100%, 85.7% and 100% for the geriatrics subgroup.

*By Bodyparts*

We compared ROC-AUC values across bodypart groups on the overall population using the unpaired DeLong test. The AI ROC-AUC was 0.970 (0.957 – 0.982 - 95% CI) for the upper limbs subgroup; 0.895 (0.834 – 0.955 - 95%CI) for the thorax subgroup; 0.933 (0.907 – 0.959 - 95%CI) for the lower limbs subgroup and 0.934 (0.887 – 0.981 - 95% CI) for the multiple bodypart subgroup.

There was no significant difference in AI ROC-AUC value across all bodypart subgroups: upper limbs vs. chest (p = 0.018); upper limbs vs. lower limbs (p = 0.012); upper limbs vs. multiple bodypart (p = 0.149); lower limbs vs. chest (p = 0,253); lower limbs vs. multiple bodypart (p = 0.977); multiple bodypart vs. chest (p = 0.317). P value for significance was set to 0.008 after Bonferroni corrections.

*By healthcare professional*

AI performance was compared to emergency physicians’ one following the protocol suggested by Roldan-Nofuentes in 2020 ^12^. Therefore accuracies (Sensitivity and Specificity) were compared on the overall population using the paired Wald test. Estimated sensitivity of emergency physicians was 93.7% (91.5% - 95.4%). Estimated sensitivity of AI was 92.9% (90.6% - 94.7%). Estimated specificity of emergency physicians was 88.5% (86.5% - 90.2%). Estimated specificity of AI was 85.6% (83.4% - 87.5%). There was no significant difference in accuracies (Se and Sp), p = 0.105. However, the estimated probability of committing type II error was 46.2% suggesting an insufficiently powered analysis.

**Supplemental references**

21.  Yu H, Chen C, Du X, Li Y, Rashwan A, Hou L, Jin P, Yang F, Liu F, Kim J, Li J

TensorFlow Model Garden. <https://github.com/tensorflow/models>. Published 2020. Accessed August 20, 2022

22. R Core Team.

R: A language and environment for statistical computing. R Foundation for Statistical Computing, Vienna, Austria. [https://www.R-project.org/](https://www.r-project.org/). Accessed September 12, 2022

23. Robin X, Turck N, Hainard A, Tiberti N, Lisacek F, Sanchez JC, Müller M,

Display and Analyse ROC Curves. <https://cran.r-project.org/web/packages/pROC/pROC.pdf>. Published 2021. Accessed September 12, 2022

***Supplemental Figure Legend***

**Supplemental Figure 1 : ROC-AUC by age subgroup.**

In orange is the overall ROC-AUC. There were no significant differences across age subgroups.

Note: ROC-AUC = area under the curve-receiver operating characteristic

***Supplemental Table***

**Supplemental Table 1 : Detailed composition of bodypart subgroups for overall and discordant patients.**

ROC-AUC of the AI model is given for the overall population. There were no significant differences across bodypart subgroups after Bonferroni correction.

Note: ROC-AUC = area under the curve-receiver operating characteristic

|  | Overall population (1772 patients) | |  | Discordant subgroup (172 patients) |
| --- | --- | --- | --- | --- |
| Bodypart | Observations (%) | ROC-AUC [95% CI] |  | Observations (%) |
| Upper Limbs  *Hand/Wrist*  *Elbow*  *Shoulder*  *Multiple segments*  *Arm/Humerus*  *Clavicle* | 738 (42%)  *376*  *153*  *110*  *42*  *32*  *25* | 0.970 [0.957 ;0.982] |  | 73 (43%)  *34*  *19 11 4 2 3* |
| Lower limbs  *Foot/Ankle*  *Knee*  *Pelvis/Hip*  *Leg*  *Multiple segments*  *Femur* | 804 (45%)  *409 186 106 55*  *30*  *18* | 0.934 [0.887 ;0.981] |  | 77 (45%)  *39*  *17 8 7 6 0* |
| Thorax  *Chest*  *Costal* | 130 (7%)  *107*  *23* | 0.895 [0.834 ;0.955] |  | 11 (6%)  *10*  *1* |
| Multiple bodyparts | 100 (6%) | 0.934 [0.887 ;0.981] |  | 11 (6%) |
